# Compliance of volunteers in a fully-enclosed patient rotation system for MR-guided radiation therapy: a prospective study

**DOI:** 10.1186/s13014-024-02461-2

**Published:** 2024-06-08

**Authors:** Cedric Beyer, Katharina Maria Paul, Stefan Dorsch, Gernot Echner, Fabian Dinkel, Thomas Welzel, Katharina Seidensaal, Juliane Hörner-Rieber, Oliver Jäkel, Jürgen Debus, Sebastian Klüter

**Affiliations:** 1 Department of Radiation Oncology, Heidelberg University Hospital, Im Neuenheimer Feld 400, 69120 Heidelberg, Germany; 2 Heidelberg Institute of Radiation Oncology (HIRO) and National Center for Radiation Research in Oncology (NCRO), Heidelberg, Germany; 3 National Center for Tumor Diseases (NCT), Heidelberg, Germany; 4 Department of Medical Physics in Radiation Oncology, German Cancer Research Center (DKFZ), Heidelberg, Germany; 5 Clinical Cooperation Unit Radiation Oncology, German Cancer Research Center (DKFZ), Heidelberg, Germany; 6 Department of Radiation Oncology, Heidelberg Ion-Beam Therapy Center (HIT), Heidelberg University Hospital, Heidelberg, Germany

**Keywords:** Patient rotation, MRI, Patient positioning, MR-guided radiotherapy, Particle therapy

## Abstract

**Background:**

Particle therapy makes a noteworthy contribution in the treatment of tumor diseases. In order to be able to irradiate from different angles, usually expensive, complex and large gantries are used. Instead rotating the beam via a gantry, the patient itself might be rotated. Here we present tolerance and compliance of volunteers for a fully-enclosed patient rotation system in a clinical magnetic resonance (MR)-scanner for potential use in MR-guided radiotherapy, conducted within a prospective evaluation study.

**Methods:**

A patient rotation system was used to simulate and perform magnetic resonance imaging (MRI)-examinations with 50 volunteers without an oncological question. For 20 participants, the MR-examination within the bore was simulated by introducing realistic MRI noise, whereas 30 participants received an examination with image acquisition. Initially, body parameters and claustrophobia were assessed. The subjects were then rotated to different angles for simulation (0°, 45°, 90°, 180°) and imaging (0°, 70°, 90°, 110°). At each angle, anxiety and motion sickness were assessed using a 6-item State-Trait-Anxiety-Inventory (STAI-6) and a modified Motion Sickness Assessment Questionnaire (MSAQ). In addition, general areas of discomfort were evaluated.

**Results:**

Out of 50 subjects, three (6%) subjects terminated the study prematurely. One subject dropped out during simulation due to nausea while rotating to 45°. During imaging, further two subjects dropped out due to shoulder pain from positioning at 90° and 110°, respectively. The average result for claustrophobia (0 = no claustrophobia to 4 = extreme claustrophobia) was none to light claustrophobia (average score: simulation 0.64 ± 0.33, imaging 0.51 ± 0.39). The mean anxiety scores (0% = no anxiety to 100% = maximal anxiety) were 11.04% (simulation) and 15.82% (imaging). Mean motion sickness scores (0% = no motion sickness to 100% = maximal motion sickness) of 3.5% (simulation) and 6.76% (imaging) were obtained across all participants.

**Conclusion:**

Our study proves the feasibility of horizontal rotation in a fully-enclosed rotation system within an MR-scanner. Anxiety scores were low and motion sickness was only a minor influence. Both anxiety and motion sickness showed no angular dependency. Further optimizations with regard to immobilization in the rotation device may increase subject comfort.

## Background

Particle therapy with protons or heavier ions makes a noteworthy contribution in the treatment of tumour diseases [1, 2]. Due to the physical nature of the dose deposition, some indications can be successfully treated using single fixed horizontal or tilted beamlines, while for other indications, the use of rotating gantries is necessary. These structurally complex, large and costly systems enable irradiation from multiple different beam angles, up to 360° rotation. In this respect, it has been hypothesized that gantries can be omitted by rotating the patient in front of fixed-beam nozzles [3, 4]. First partially-enclosed prototype devices for patient rotation have already been investigated in small scale to test feasibility and imaging capabilities [5–9]. While imaging in particle therapy today usually is performed with x-ray-based systems, also the use of magnetic resonance imaging (MRI) has become increasingly important [10–12]. In photon radiotherapy, integrated hybrid-systems combining MRI and linear accelerators are already well-established [13–18], and first concepts for combining particle therapy with MRI have been evaluated [19–21].

However, the use of patient rotation devices may be specifically challenging when combined with magnetic resonance (MR) imaging, as anxiety and claustrophobia are already of concern in conventional MRI [22–28] and might be even further enhanced with the use of partially or fully-enclosed rotation systems. Also, the general patient well-being and comfort pose apparent obstacles in the implementation of these patient immobilization systems into clinical treatments. In this manuscript, a fully-enclosed patient rotation system was used to quantify the compliance of volunteers to immobilization and rotation under MRI.

## Materials and methods

### Patient rotation system

**Fig. 1:**
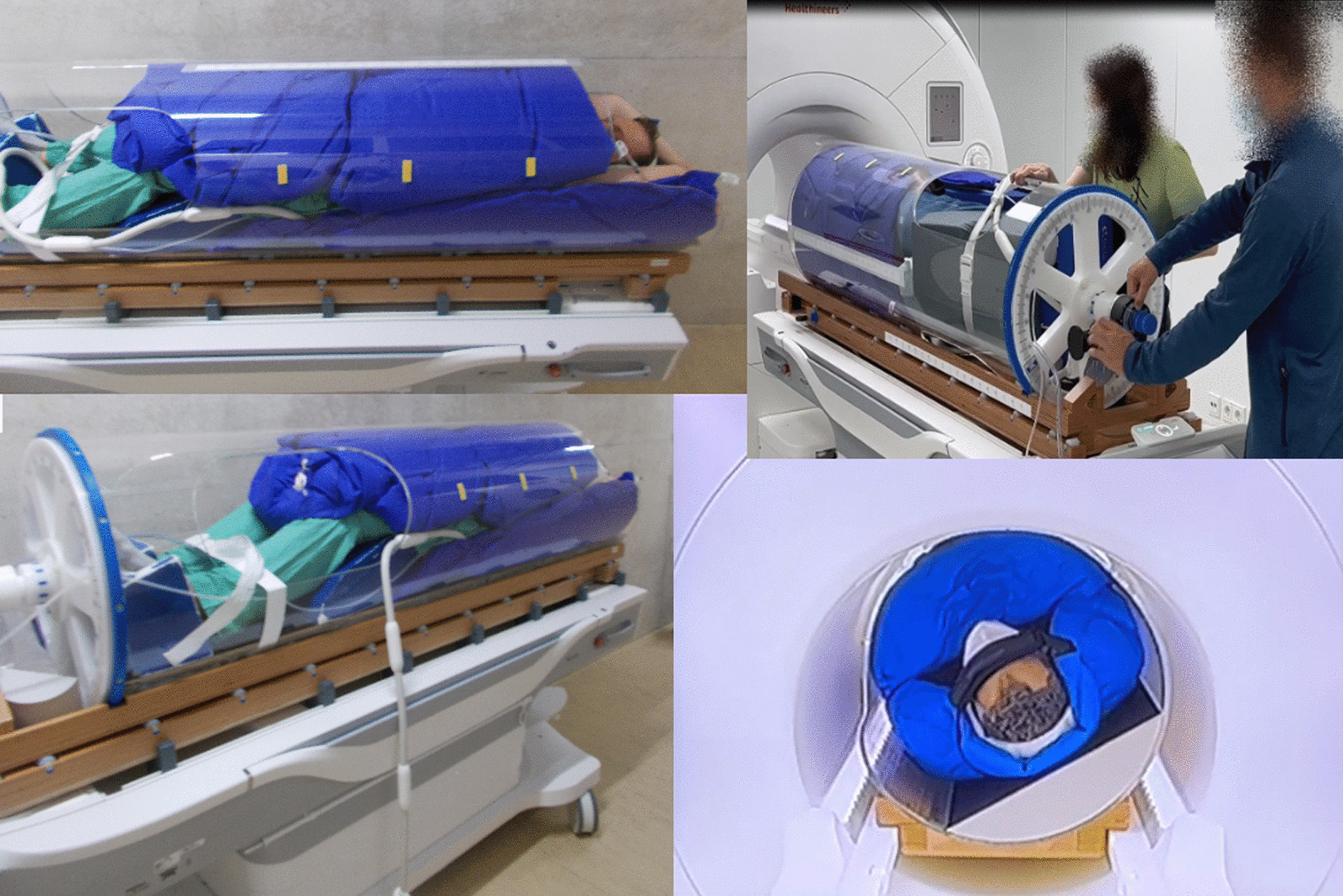
Patient rotation system with subject in different positions during the study

A fully-enclosed patient rotation system (PRS) for the reproducible positioning of a previously immobilized patient for a diagnostic or radio-therapeutic procedure in supine, prone or any rotated position was developed. The rotation system can be positioned in front of a horizontal treatment beam or placed in an MRI or CT with bore diameters of at least 700 mm. A patient within the PRS can be rotated around its longitudinal axis in order to realize different imaging positions and beam entry angles, respectively. The PRS consists of a polymethyl methacrylate tube (length: 2250 mm, inner diameter: 500 mm). The cylindrical, fully-enclosed design was chosen so that no inhomogeneities are to be expected in the beam path, thus ensuring transmissivity without problematic boundaries, especially for potential irradiation with particles. An MR-imaging-coil can be easily integrated by placing thin conductors on the cylinder surface like suggested by Dietrich et al. [29, 30]. The tube rests on a wooden foundation and can be manually rotated, either with a geared handle or, after decoupling from the gear, fully manual (Fig. 1). The foundation can be moved transversely on a base plate. The base plates provide the fixation for the tables of the diagnostic and therapeutic modalities. For a more detailed, technical description of the system the reader is referred to Echner et al. [31].

### Study design

The study with a total of 50 adult volunteer participants (33 m; 17 f) was divided into two phases. The first phase (subjects #1-20) consisted of a simulation of an MR-acquisition, the second phase (subjects #21-50) of real MR-imaging. The subjects were positioned either with their arms along the body (subjects #1-35) or overhead (subjects #36-50). All subjects could terminate the study at any time and without giving any reason. The basic assumption was that less than 20% of the subjects would drop out of such a study with a fully-enclosed patient rotation system. Right after the informed consent interview, a claustrophobia questionnaire (CLQ) after Radomsky et al. [32] was filled out, and age, height, weight, shoulder width, hip circumference, and abdominal circumference were determined. This served as a reference for claustrophobia. The immobilisation technique, which was common in both phases, involved the use of vacuum mats. A first vacuum mat served as a base and ranged from the head up to and including the hips (Fig. 1). A second vacuum mat was used to immobilise the front of the subject from the shoulders to the navel. Two 18-channel flex-coils (Body 18 long Tim coil 1.5T, Siemens Healthineers, Erlangen, Germany) were inserted between the subject and each of the vacuum mats in the front and back. A third vacuum mat immobilised the subject anteriorly from the navel to the knees. Subjects were immobilised either in the rotation system or outside with the help of a mould for vacuum mats. If the immobilisation took place outside, the immobilized subject was pushed into the rotation system via the front opening of the PRS. During the simulation (phase 1), the immobilised subjects were placed in a 1.5 Tesla Magnetom Sola^©^ (Siemens Healthineers, Erlangen, Germany) in the PRS at 0°, 45°, 90° and 180° rotation angles and MR-pulse-sequence audio sounds were played through the headphones. For each angle setting Gradient echo sequence (GRE), echo-planar fast spin echo sequence and cine balanced steady-state free precession (bSSFP) sequence audio sounds were played for a total playing time of 10 min. Rotational speeds were measured and recorded once for 5 subjects during simulation. For that, the time was stopped which was necessary to rotate the subject from a 180° to 0° and an average speed was calculated. Anxiety was assessed using a 6-item State-Trait-Anxiety Inventory (STAI-6), for motion sickness a modified Motion Sickness Assessment Questionnaire (MSAQ) was used. Motion sickness was assessed directly after each rotation and anxiety after simulation or acquisition at the respective angle. For this purpose, the head of the subject in the capsule was approached and in direct conversation they had to state the extent to which they agreed with the questions of the questionnaires according to the metrics described in “Assessment and evaluation” section. Furthermore, any additional areas of complaint were assessed.

### Image acquisition

For the imaging phase (subjects #21-50), the existing simulation setup was adapted to a scenario, which could be, in theory, used for an irradiation of the liver, pancreas, the retroperitoneal space or the lower thoracic or lumbar region. For this purpose, instead of playing back audio files, real MRI-acquisition was performed and the angles were adjusted accordingly to 0°, 70°, 90° and 110°. To further avoid a possible bias or misinterpretation of the tolerability of the measurement angles based on their running order, this order was randomised for every subject. For imaging, a T1 3D GRE (TE: 2.39 ms/4.77 ms; TR: 6.9 ms; voxel size: 1 × 1 × 5 mm^3^; FOV: 500 × 500 × 320–420 mm^3^; duration: 23 s), a spiral ultra-short echo GRE (0.05 ms; 3.5 ms; 1.7 × 1.7 × 3 mm^3^; 700 × 700 × 320–420 mm^3^; 20 s) and a T2 CINE bSSFP (1.52 ms; 130.3 ms; 1 × 1 × 7 mm^3^; 500 × 500 × 7 mm^3^; 92 s) sequence were performed. The imaging field-of-view extended from the liver to the iliac crest. Anxiety was assessed directly after every scanned angle. Motion sickness was assessed directly after every rotation. After imaging had been performed at all angles, the imaging was repeated at all angles in the same running order without assessing anxiety or motion sickness. After that, the subjects were removed from the MRI and the PRS rotation system. To assess the quality of the immobilisation and position accuracy both inter- and intra-fractionally, the subjects were reintroduced into the system and the MRI a second time and images at all angles were acquired for a third time. The subject's state of mind and condition were permanently monitored and ensured by non-standardised questioning. Rotational speeds were measured and recorded once for eight subjects during imaging. This measurement took place at the rotation between the last angular position of the first run and first angular position of the second run. This was due to the fact that the running order of the angular positions was random. In total, 10 position sets with 10 min imaging duration each were utilized. Thus, the time the subject remained in the capsule was 100 min, with a 10-min break after 70 min. The recorded image data and survey results were stored in a database.

### Assessment and evaluation

The claustrophobia questionnaire (CLQ) was evaluated using the methodology published by Radomsky [32] and Napp et al. [33]. Here, the subjects had to assess their fear in 26 situations. The scale for the individual items can range between 0 (no fear) and 4 (extreme fear) in whole-number increments. Then the average claustrophobic fear score per statement and the relative level of agreement in percent over all items combined is determined. A result of 0% shows no anxiety, whereas 100% represents the maximum possible intensity for claustrophobic anxiety.

Anxiety was assessed with a 6-item State-Trait-Anxiety Inventory (STAI-6) according to the methodology published by Marteau et al. [34]. Within the STAI-6, the subjects have to answer 6 statements about their condition at time of assessment. The scale for the individual items can range in whole-number increments between 0 (not at all true) and 4 (very true). Then the percentual level of agreement over all 6 items within a position was determined. A result of 0% shows no anxiety, whereas 100% represents the maximum possible intensity for anxiety.

The assessment and evaluation of motion sickness was based on a modified 16-item Motion Sickness Assessment Questionnaire (MSAQ) by Gianaros et al. [35]. The modification of the MSAQ was an exclusion of all experience statements not affiliated to gastro-intestinal, central or peripheral symptoms. The subjects had to answer 12 statements about the presence and severity of symptoms at the time of the assessment. The scale for the individual items can be in whole-number increments between 0 (no symptom) and 20 (extreme symptom intensity). Then the relative level of agreement in percent over all items within a rotation was determined.

Evaluation of the questionnaires was performed using an in-house developed Python script. Spearman correlation coefficients were determined and Wilcoxon signed-rank tests and Mann–Whitney U tests were performed. The latter two involved the null hypothesis that there were no differences between the compared variables, and a *p* value below 0.05 was considered statistically significant. Additionally, the individual complaints were collected, grouped by body region and presented as a percentage of the total number of complaints submitted for a given angle position.

## Results

**Fig. 2:**
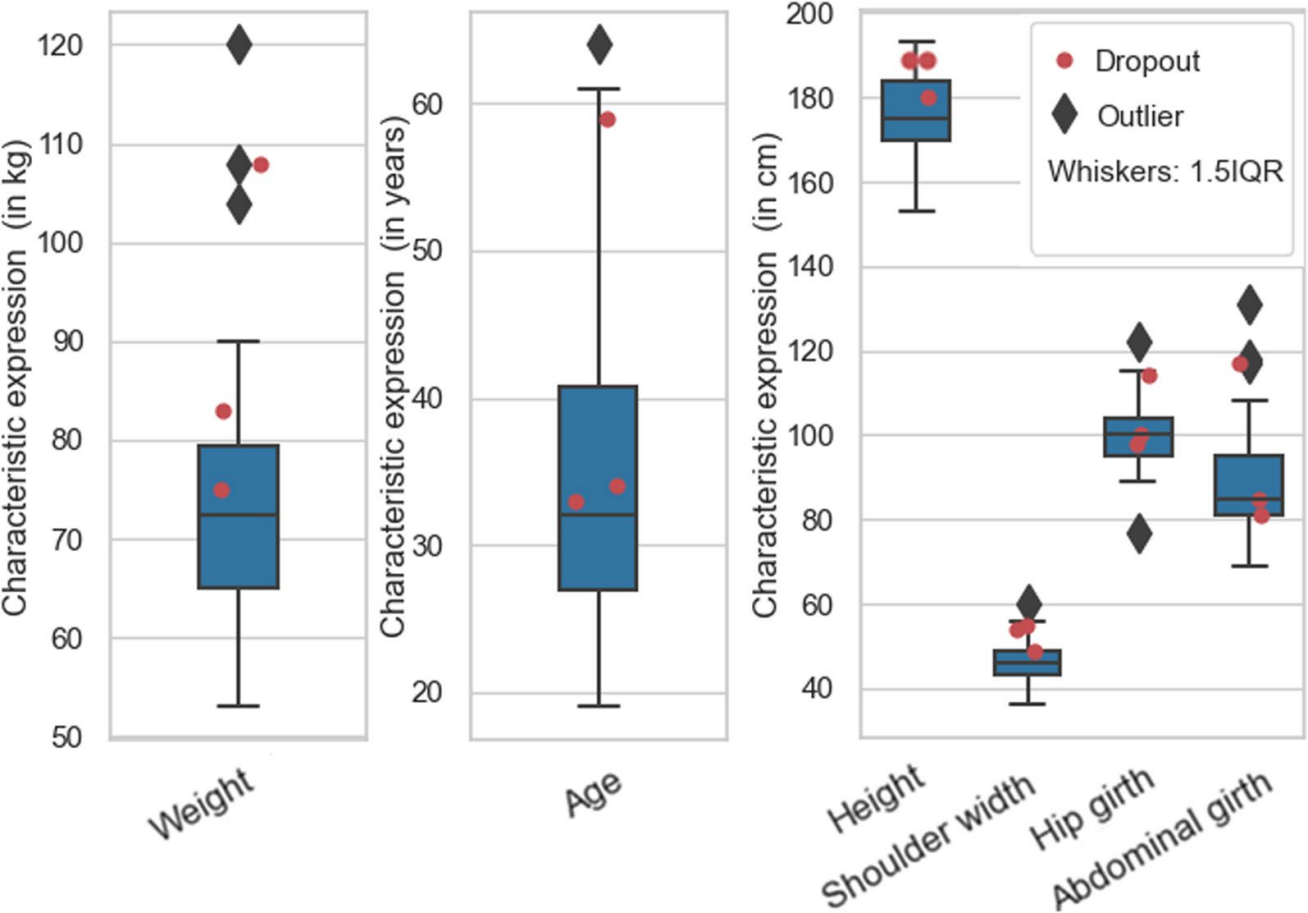
Quantified body parameters of all subjects; red dots mark the dropouts

For the study a diverse collective of subjects could be acquired. Figure 2 shows the body proportions of the subjects. Average scores on the CLQ per statement were 0.64 ± 0.33 (16.75 ± 8.57%) for the simulation cohort and 0.51 ± 0.39 (12.93 ± 10.18%) for the imaging cohort. For the combined cohorts the average CLQ-score per statement was 0.56 ± 0.39 (14.5 ± 9.8%).

**Fig. 3:**
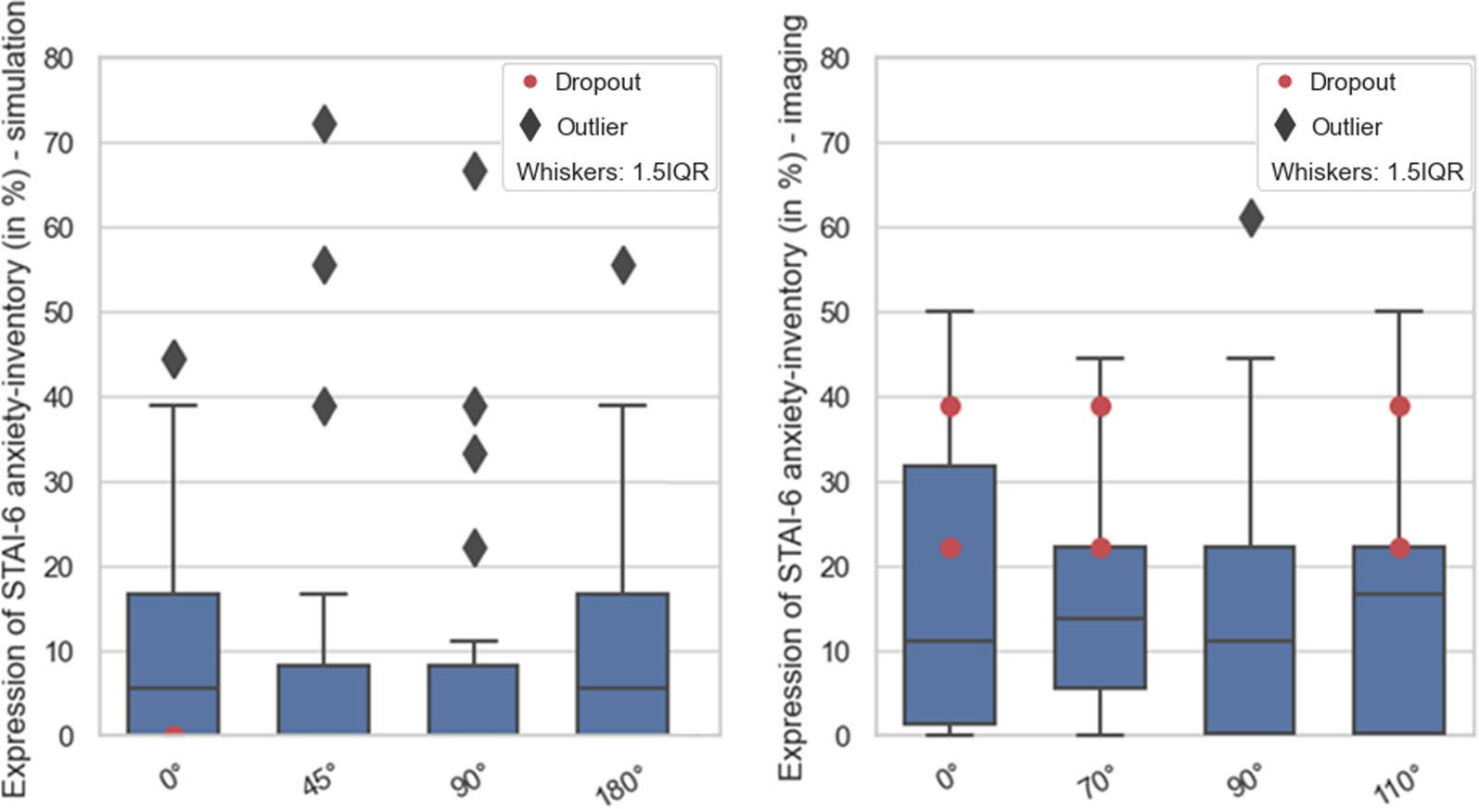
Normalised anxiety score for simulation and imaging cohort. Red dots mark the dropouts

**Fig. 4:**
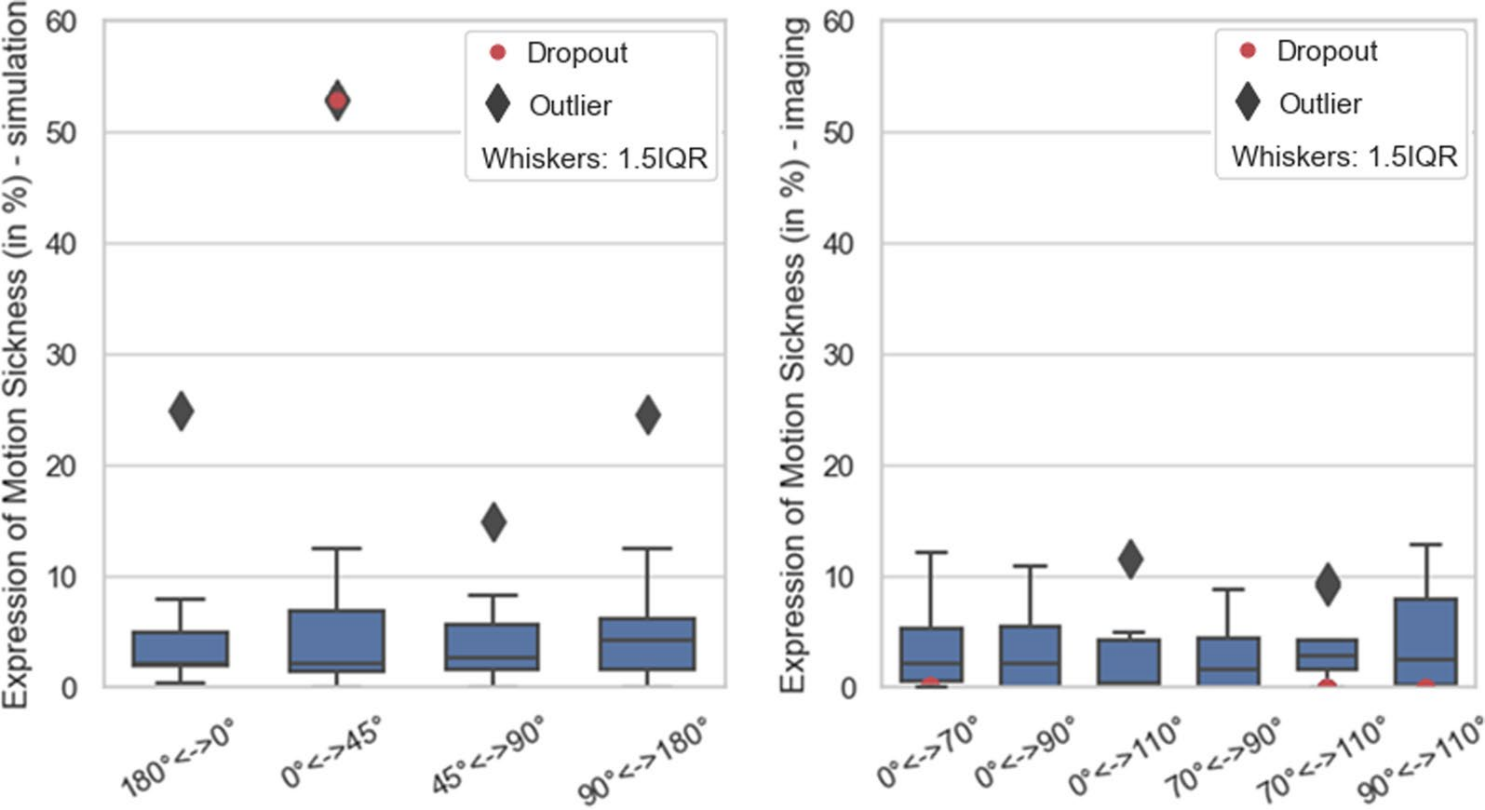
Normalised Fast motion sickness scores for simulation and imaging cohort. Red dots mark the dropouts. Patients with scores = 0 were excluded

STAI-6 anxiety at different rotation positions is shown in Fig. 3 and motion sickness at different rotations in Fig. 4. Some degree of motion sickness was present in 75% (simulation) and 37% (imaging) of all subjects. The further statistical evaluation of CLQ and STAI-6 is displayed in Table 1a, b. No statement could be made about monotonic correlation between claustrophobia and anxiety with the used level of significance of 5% (*p* ≥ 0.108). Between the anxiety inventories for different angles presented in Table 1a, b, a moderate monotonic correlation, with a significance level of 10%, can be drawn between the measurement angles equal to and unequal to 0°. The measurement angles other than 0° showed a strong monotonic correlation with a significance level of 5%. Both was valid for simulation and imaging. The two-sided Wilcoxon Signed-Rank Test displayed in Table 1c proved equality of anxiety between all angular positions to a level of significance of 5%. At a significance level of 5%, the Man-Whitney U test (see Table 2) provided no sufficient statistical evidence for a significant difference of motion sickness at different rotations.

**Table 1:** Statistical evaluation of STAI-6 and CLQ in different angles. All analyses excluded the dropouts per protocol

Simulation	STAI 0°	STAI 45°	STAI 90°	STAI 180°
(a) Spearman correlation coefficients of subjects during the simulation with *p* values (correlation/*p* value)				
CLQ	− 0.25/0.294	− 0.12/0.952	− 0.14/0.546	− 0.09/0.695
STAI 0°		0.53/0.019	0.44/0.057	0.45/0.056
STAI 45°			0.88/ < 0.001	0.64/0.003
STAI 90°				0.73/ < 0.001

Imaging	STAI 0°	STAI 70°	STAI 90°	STAI 110°
(b) Spearman correlation coefficients of subjects during MRI with *p* values (correlation/*p* value)				
CLQ	0.31/0.108	0.22/0.257	0.13/0.505	0.04/0.810
STAI 0°		0.67/ < 0.001	0.5/0.005	0.49/0.008
STAI 70°			0.75/ < 0.001	0.75/ < 0.001
STAI 90°				0.7/ < 0.001

Simulation	STAI 45°	STAI 90°	STAI 180°	Imaging	STAI 70°	STAI 90°	STAI 110°
(c) *p* values of Wilcoxon signed-rank test (two-sided) for subjects during MRI							
STAI 0°	0.609	0.721	0.812	STAI 0°	0.319	0.600	0.513
STAI 45°		0.395	0.674	STAI 70°		0.878	0.875
STAI 90°			0.553	STAI 90°			0.727

**Table 2:** Statistical evaluation of motion sickness in different angles. All analyses excluded the dropouts per protocol

Simulation	45 → 90	90 → 180	180 → 0
(a) *p* values of motion sickness comparing different rotations for the simulation cohort (Mann–Whitney-U test)			
0 → 45	0.965	0.661	0.857
45 → 90		0.643	0.786
90 → 180			0.468

Imaging	0 ↔ 90	0 ↔ 110	70 ↔ 90	70 ↔ 110	90 ↔ 110
(b) *p* values of motion sickness comparing different rotations for the imaging cohort (Mann–Whitney-U test)					
0 ↔ 70	1.0	0.712	0.712	0.857	0.934
0 ↔ 90		1.0	0.951	0.844	0.685
0 ↔ 110			1.0	0.628	0.685
70 ↔ 90				0.389	0.607

The rotation speeds at which the subjects were rotated were 38.6 ± 15.9°/s (simulation) and 58.1 ± 32.3°/s (imaging).

During simulation, one subject dropped out in the first run due to nausea when rotating to 45°, while during imaging, two subjects dropped out in the first run at 90° and 110° (3rd and 4th measurement angle, respectively) due to pain from positioning. The time remaining in the rotation system up to this point was 30 and 40 min, respectively. In total, out of 50 subjects, only 3 (6%) subjects terminated the study prematurely.

**Fig. 5:**
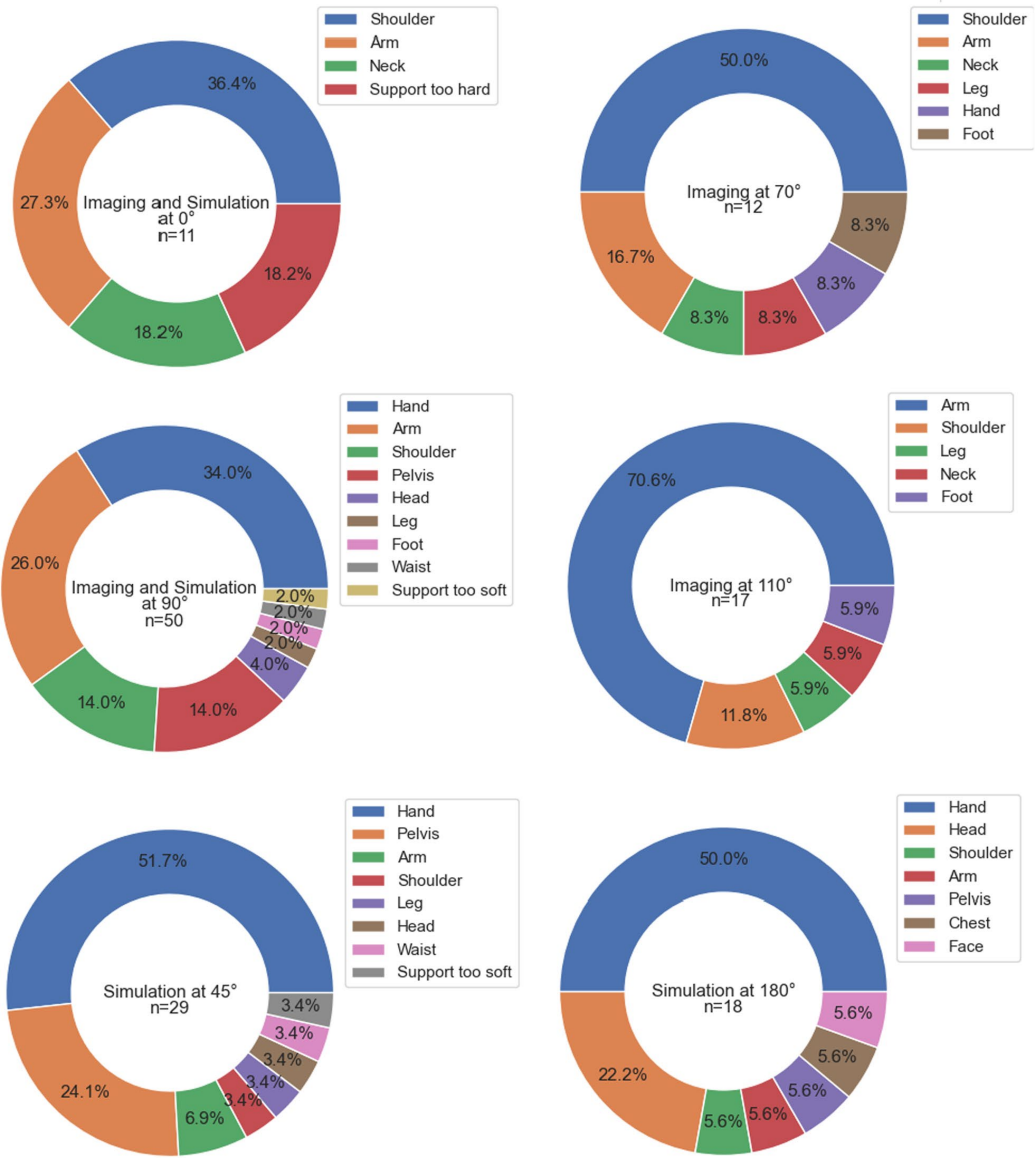
Reported areas of discomfort for all participating subjects. Percentages mark the relative to the total amount of complaints filed for that angle. Filing multiple complaints was possible

In Fig. 5, all qualitatively recorded comfort-related complaint regions are listed. Main factors triggering complaints were pressure from the subjects’ own body weight on the shoulders and extremities, unpleasant folds present in the vacuum mats and a tightness caused by the immobilisation that exerted pressure on the blood circulation and nerves.

Apart from the reported immobilization discomfort, the feedback of the subjects was positive in terms of feasibility. The setup time of up to 15–20 min was well tolerated. After setup completion, the reduction in comfort started, and discomfort and the subsequent occurrence of pain points increased.

**Fig. 6:**
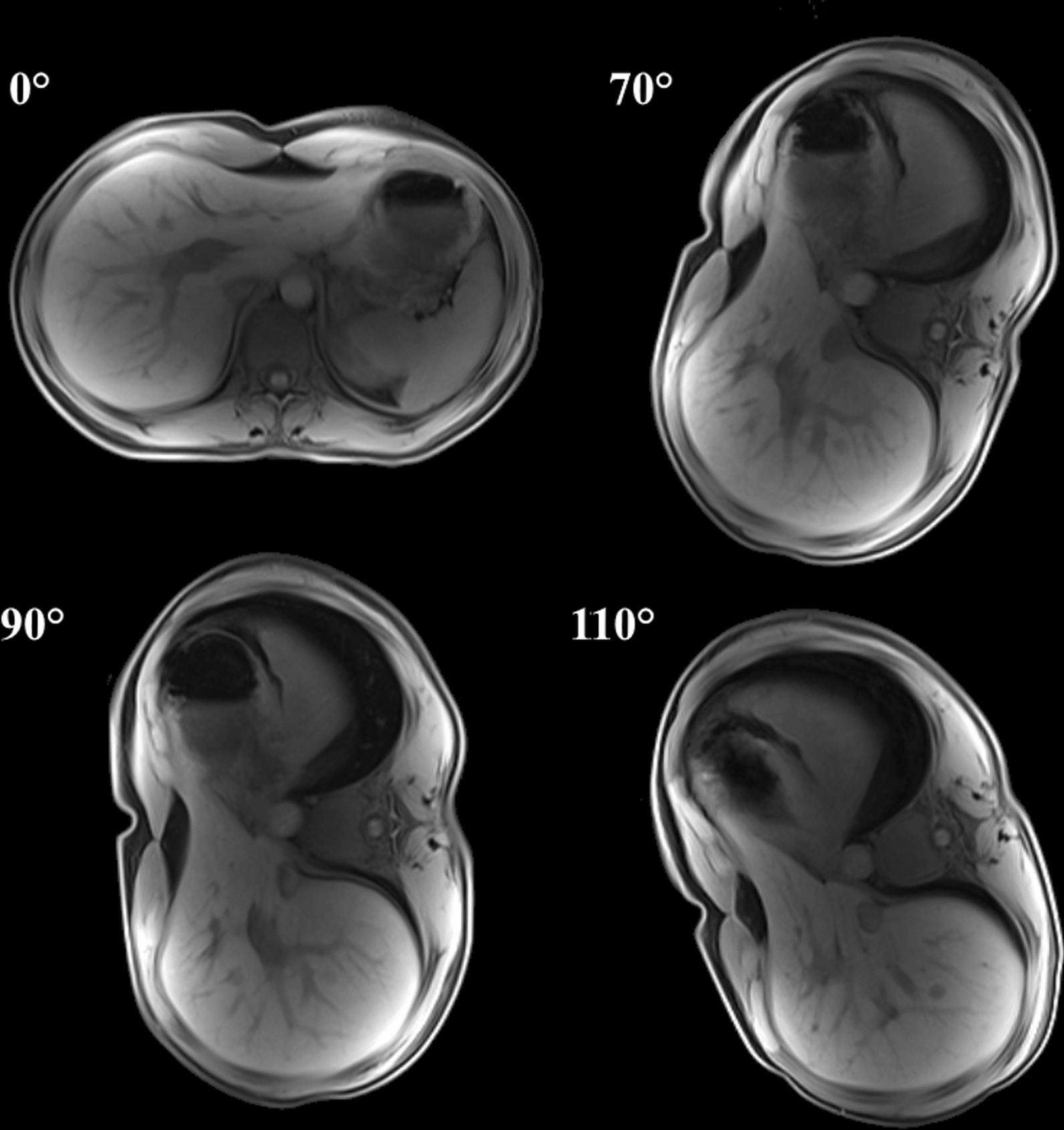
T1-GRE images of a subject at the same axial position in different rotation statesable

As an example of the acquired MR-images, Fig. 6 shows axial slices of the T1 GRE scan of one subject at the four different rotation angles.

## Discussion

In this article, we present a study on the compliance of healthy subjects in a fully-enclosed patient rotation system for MR-guided particle therapy. Only three of the 50 participants aborted the study, thus confirming the basic assumption that less than 20% of the subjects would drop out of such a study with a fully-enclosed patient rotation system (*p* = 0.0057). Further, the study showed a good acceptance of the rotation system in realistic conditions across all rotation angles for use on subject populations without a tendency to claustrophobic events. Anxiety and especially motion sickness were present but only occurred with negligible intensity. The monotonic correlations involving claustrophobia can most likely be explained by randomness due to low correlation coefficients and the subsequent monotony and high *p* values. This is a strong evidence that claustrophobia is not linked to the different rotation positions. The results of the Wilcoxon-signed rank test underline that anxiety is not related to specific angles, rather the overall duration and general positioning seem to influence the tolerance of the subjects. The measurement results further showed no decrease in patient compliance for specific measurement angles. The compliance of the participants however depends on body parameters like shoulder width and body height, especially in relation to other sensitivities of the subjects. A connection between study dropout and increased anxiety or increased motion sickness cannot be drawn. The dropouts showed levels of anxiety which are higher than the median of their individual cohort. This was caused by low levels of comfort. The other sensitivities showed almost exclusively uncomfortable feelings of different body regions, primarily the shoulder and upper extremities. We didn’t find any difference in overall comfort for different arm positioning (along the body or arms overhead), leading to the same complaints in both cases, caused by general tightness and burdening of the extremities with one's own body weight**.**

The presented results do not indicate any significant effects of motion sickness for this application of the rotation system. However, the results showed that a higher motion sickness was reported in the simulations compared to the imaging. While the simulations were conducted in summer and the imaging part of the study took part in winter, this might have an impact on the overall motion sickness as the peripheral symptoms of motion sickness, expressed as warmth/heat and mischievousness were also asked for within the survey of motion sickness. This might have influenced the corresponding results of motion sickness and shows an area for optimization of the rotation system.

Overall, our study indicates a positive assessment of the suitability of fully-closed patient rotation systems for future clinical applications. Advantages of these systems are the high homogeneity of the wall material around the patient to facilitate therapy planning while having 360° access for beam delivery. Direct disadvantages, in contrast to partially-closed systems, apply only during immobilization of the patient, where the patient is not as accessible in a fully-closed system.

Regardless of the design, adjustments should be made to increase patient comfort and usability in follow-up studies. Our subjects complained about comfort and heat accumulation of the immobilization material on multiple occasions. Future studies should therefore focus on different immobilization materials than the vacuum matrasses used here.

In the long term, it cannot be relied upon that the MRI bore-diameters will further increase which would allow for larger rotation systems with improved patient comfort. Open MRIs would in that regard be beneficial for in-beam use because this would give room for elliptical shapes of rotation devices, which might give more room for extremities and people with wide bodies.

Based on literature, a distinction between population-related differences and superiority or inferiority of the performed rotations and positions in the patient rotation system is not possible. The studies of Napp et al. [33] and Harris et al. [23] performed only MR-imaging without a rotation system. The average CLQ scores of 0.60 ± 0.5 presented by Napp et al. [33] for a study population without a tendency to claustrophobic events are comparable with our findings. Similarly to our procedure Harris et al. [23] acquired the anxiety inventories directly after the scan. Their results of this general study of anxiety during MRI-examinations (CLQ scores 20.4 ± 18.3 male; 35.5 ± 23.0 female), showed greater discrepancy with our results. The discrepancy is caused, presumably, by their patient collective being more prone to anxiety in general. These comparisons suggest that the main anxiety factor claustrophobia, during measurement, is not impacted negatively by the MRI-rotation-system.

Whelan et al. [4] assessed anxiety and motion sickness of patients rotated within a gyroscope. They reported a mean CLQ score of 19 ± 20 (normalized values), corresponding well with our results. Also, their mean STAI scores of 11.8 match our findings, which suggests that the narrower rotation system used in our work does not have an influencing effect on anxiety. Whelan et al. [4] reported, however, a mean motion sickness score of 13.7, which is increased compared to our results. Their claim of a median motion sickness score of 0 is equal to our findings. This suggests general consistency and indicates that the used methodology for motion sickness assessment is sufficient. Rotation in a narrower rotation system, compared to an open gyroscope, has no negative impact to motion sickness. The differences especially in the mean motion sickness are, probably, due to the fact that the collective of Whelan et al. [4] was slightly more prone to motion sickness in single individuals.

Buckley et al. [5] performed MR-imaging in a partially enclosed rotation system with increments of 45° over a 360° rotation with patients and subjects. Prior to and after imaging anxiety and motion sickness was assessed. There, 3 of the 20 subjects (15%) did not complete the study. The dropout rate presented in our work was comparable, but slightly less. The data collected by Buckley et al. [5] showed also further similarities, although they didn’t record each individual rotation angle and only a before and after comparison is available. In their study 55% of participants showed no motion sickness and also for the set-up presented, there are no discernible differences in the strength of the anxiety and motion sickness [5]. Nevertheless, it can be concluded that motion sickness is negligible during patient rotation and in combination with MR-imaging no factor for additional anxiety based on the positioning in a fully-enclosed rotation system is present.

Buckley et al. [5] reported also on sensitivities beyond anxiety or motion sickness. The complaints reported there also targeted the extremities. However, this only applied to the arms and shoulders when the arm position was above the head. This is a common problem among medical procedures and interventions, also outside of radiotherapy, which require the arm being out of certain areas around the body. They also reported isolated problems due to pressure on the thorax area which could not be observed within our study. This underlines that comfort is related to the immobilization technique as well as individual conditions.

Due to the voluntary nature of our study, participants with a tendency to claustrophobia, increased anxiety or panic attacks had the option of not responding to or dismissing the call for participation. Therefore, the participants had no tendency towards claustrophobia and maybe increased anxiety. On the other hand, it can be assumed that cancer patients will show a higher tolerance during a medical intervention that might prolong their life or have pain-fighting effects. This could therefore result in an increased acceptance of a rotation system and immobilization in a patient cohort. Although the literature on anxiety levels for radiation therapy procedures is sparse, some reports support this assumption. For a cohort of patients who underwent radiological investigations, Lo Re et al. [36] found that patients with an oncological disease showed lower levels of anxiety than patients with no oncological diseases. A recent study of Moreira et al. [37] found low anxiety levels in patients treated with a 1.5T MR-Linac. Antoni et al. [38] evaluated anxiety in breast cancer patients and reported that roughly a quarter of all patients showed clinically relevant levels of anxiety, but this number showed a tendency to decrease already after initial treatment planning. Overall, while only a follow-up study with patients can definitely answer the question if cancer patients will tolerate rotation in a fully-enclosed rotation system, our results build an important foundation such a study.

In order to obtain more detailed information about areas in need of optimization, it is advised to conduct follow-up studies focusing on usability as well as improving comfort, also looking at detailed areas of discomfort at different body parts. Follow-up studies with different methods of patient immobilization in the rotation system shall also be advised, to limit the immobilization effort. As already pointed out, these should be conducted with volunteer patients rather than healthy volunteers in order to gain insights into collectives with additional comorbidities or higher anxiety levels. The assessment, incorporating organ movement for different rotated patient positions into particle treatment planning will be important for clinical introduction of patient rotation systems. We are already in the process of analyzing the acquired data in that sense and will conduct treatment planning studies accordingly.

Furthermore, an accurate quantification of the positioning qualities and the positional reproducibility using a rotational device will be a necessary prerequisite for the clinical introduction of such systems. The questions regarding the differential impact of rotation on particle dose deposition in various anatomical states are of high significance and have to be addressed in future studies to ensure safe and effective clinical deployment. Therefore, it is imperative for subsequent research to explore the nuanced effects of rotation on different patient populations when adequate reproducibility and position quality of such systems is achieved. Efficient ways of treatment planning will need to be developed in order to deal with different internal anatomy following rotation of a patient, most likely by use of deformable image registration and dose accumulation. Also, with changing internal anatomy following rotation, optimal angles might be different from currently used gantry angles for specific indications, and this will also need to be evaluated in future studies using real patient data. Projects are currently ongoing at our institution in order to find solutions for these challenges, with the aim of being able to use the rotation device for clinical particle beam treatments eventually.

Lastly, the integration of necessary MR-equipment within the rotation system should be increased. For example, the coils required for the MR-measurement could be directly integrated within the rotation system as a transmit- and receive-coil, as already simulated, conceptualized and tested by Dietrich et al. [29, 30]. This would make additional receiving coils directly around the patient obsolete and lead to easier positioning and immobilization of the patient.

## Conclusion

A fully-enclosed patient rotation system was used to quantify the compliance of subjects to immobilization and rotation under MRI. Claustrophobia as well as anxiety was found to be negligible. Anxiety was independent to angular position. Motion sickness was negligible as well and equal between all rotations performed. Enhancement of patient comfort was identified as one of the primary areas of concern and future studies should focus on patients with oncological diagnosis.

## Data Availability

The datasets generated and/or analyzed during the current study are not publicly available due General Data Protection Regulation (GDPR) but are available from the corresponding author on reasonable request.

## Abbreviations

CINE: Steady-state free precession (SSFP) gradient echo-imaging
CLQ: Claustrophobia questionnaire
CT: Computer tomography
FOV: Field-of-view
GRE: Gradient echo sequence
HASTE: Half-Fourier acquisition single-shot turbo spin echo imaging
IQR: Interquartile range
MR/MRI: Magnetic resonance/magnetic resonance imaging
MRgPT: MR-guided-particle therapy
MRgRT: MR-guided-radiotherapy
MSAQ: Motion Sickness Assessment Questionnaire
PRS: Patient rotation system
STAI-6: 6-Item state-trait-anxiety inventory
TE: Echo time
TR: Repetition time
